# A Phenomenological Study of Mental Health Enhancement in Taekwondo Training: Application of Catharsis Theory

**DOI:** 10.3390/ijerph18084082

**Published:** 2021-04-13

**Authors:** Won-Chul Bing, Soo-Jung Kim

**Affiliations:** 1 Division of Sport Science, Baekseok University, Cheonan-si 31065, Korea; bing7@bu.ac.kr; 2 Department of Taekwondo, Dong-Eui University, Busan-si 47340, Korea

**Keywords:** mental health, Taekwondo training, theory of catharsis

## Abstract

In modern society, catharsis is often understood as the relieving of stress, and the psychological and medical effects of catharsis are well known even to ordinary people. There are many studies showing that physical activity is a good tool for managing and promoting mental health. However, there are not many studies on Taekwondo training and catharsis. Therefore, we conducted a study explaining catharsis as mental health promotion in Taekwondo training. This study explores mental health enhancement of Taekwondo training by using a phenomenological methodology. Phenomenology is a theory that seeks to understand an individual’s recognition of their own subjectivity rather than explaining objective factors about an individual. We collected data from interviews with 12 students who had been members of a university Taekwondo demonstration team. The phenomenological results were expressed as six themes: (1) vicarious purgation of repressed emotions, (2) emotional catharsis through pity and fear, (3) catharsis from ethics, (4) catharsis through mimesis, (5) catharsis from vicarious satisfaction through teammates, (6) catharsis from being the object of envy. Taekwondo, a traditional Korean martial art, is a physical activity that allows people to experience catharsis, which is a mental health effect of sports.

## 1. Introduction

In modern society, many people are exposed to mental, physical, and social problems and are suffering from tremendous stress due to the complexity, diversification, and specialization of the industrial structure and other unpredictable variables; therefore, each individual or group strives to reduce the sense of burden in life through various activities. In particular, sports activities, with various positive effects on physical and mental stability and satisfaction, play the role of a haven for people living in modern society [1,2].

Many people associate sports with quantitative aspects that are externally manifested such as competition, championship, training, pain, and records. However, when discussing sports, not only these quantitative aspects, but also qualitative aspects including human emotions like joy, anger, sorrow, and pleasure need to be considered [3]. In other words, sports have both quantitative and qualitative aspects. To a sports performer, sports activities have both the quantitative meaning of bodily movement and the qualitative meaning of inner emotions created by it, regardless of the results of the activities. This study focuses on such inner emotions created by sports activities; that is, the qualitative values of sports.

Taekwondo, which debuted as an official sport at the 2000 Olympic Games in Sydney, has grown into a global sport with more than 60 million practitioners in 182 countries around the world, representing the Korean sports culture [4]. Today, it has become a global sport that has gained an international reputation, and stands among the official games in the Olympics. Taekwondo is one of the most systematic and scientific Korean traditional martial arts, that teaches more than physical fighting skills. It is a discipline that shows ways of enhancing our spirit and life through training our body and mind.

Many studies have been conducted on the value of Taekwondo as mental education. First, it has been reported that Taekwondo practice increases physical confidence, self-esteem, and respect towards peers among adolescents, and it lowers the level of their academic stress [5,6,7]. In addition, several studies have reported that Taekwondo practice can help cultivate courage, willpower, good manners, leadership, and determination, and has a positive effect on personality and etiquette education [8,9,10,11,12]. There are also studies showing that Taekwondo practice reduces the aggressive behavior and stress of the practitioners [13,14]. However, most of the preceding studies on the spiritual value of Taekwondo took a quantitative approach and exposed limitations in revealing more profound levels of value inherent in Taekwondo practice. Therefore, a qualitative study such as phenomenological study is needed to understand the relationship between Taekwondo training and mental health improvement.

People often say they experience catharsis through sports. Cathartic experiences occur through emotional immersion in sports players’ great moves and performance. People also experience catharsis when they participate and engage in sports activities. For example, a cancer patient was asked about the reason why he tried to run in a marathon in a TV interview and he replied that it gives him a cathartic experience that could sustain him during his fight against cancer. In this way, many people experience “sports catharsis” through participation in sports activities [15].

The term “catharsis” (“katharsis” in Greek) was first used by Aristotle in the sixth chapter of the Poetics to define the meaning of tragedy. Catharsis means purification in a moral sense, purification in a religious sense, and purgation in a medical sense. In psychology, it also refers to relieving repressed feelings or shock [16]. Initially, catharsis meant a phenomenon where the audience members of a play release their emotional baggage while sympathizing with the characters in a play. However, catharsis in modern society is not only related to plays, but can be experienced in various forms.

In modern society, the term catharsis is often understood as the relieving of stress, and the psychological and medical effects of catharsis are well known even to ordinary people [17].

The understanding of cathartic experience through participation in sports activities transcends quantitative and mechanistic understanding based on the positivist understanding. The excitement and pleasure, joy, and emotional purification that are experienced during sports activities can be understood with a phenomenological approach, a research methodology based on experience. This study is therefore conducted with a phenomenological research method.

In the field of sport humanities, phenomenological studies were conducted in earnest in the 1970s and 1980s [18]. American sports philosopher Kleinman (1964) published the paper “The Significance of Human Movement: A Phenomenological Approach” [19]. This research is the first phenomenological study in the field of sports, and has been the basis for phenomenological concepts and methodologies in the field of sport humanities until now [18].

Phenomenological research is an attempt to explain the essential element of physical experience from experiential perspective [20,21]. In particular, in the field of sport humanities, the essential characteristics of exercise experience and their meaning are explored [22]. Phenomenological research understands the inner side of human behavior through experiential techniques and analysis, in-depth interviews, and categorization [23,24]. Thus, because a phenomenological approach analyzes profound and personal experiences [25,26,27], the sports activities experiences can be understood subjectively.

The purpose of this study is to provide new information on the improvement of mental health in Taekwondo training. This study, which analyzed the catharsis of Taekwondo training, can confirm that physical activity is a good tool for improving mental health.

## 2. Study Methods

### 2.1. Study Design

This study explored the catharsis experience of Taekwondo demonstration team members in depth with a phenomenological research approach. The study adopted qualitative research methodology, especially phenomenological research methodology, because it was important to identify the meanings of the phenomena experienced by the study participants in exploring the study subject [28].

Most Korean colleges and universities that have Taekwondo departments operate Taekwondo demonstration teams. Students of Taekwondo departments participate in the activities of demonstration teams, gyeorugi (sparring fight) teams and pumsae (sequences of movements) teams according to their specialty. This study especially focused on the cathartic experience of Taekwondo demonstration team members as it was assumed that Taekwondo demonstration activities would represent the essential aspect of catharsis, consisting of elements such as tension, fear, failure, success, and joy based on a script.

Written consent was provided by each of the study participants. Interviews were conducted at Taekwondo halls, cafes, and labs so that the students could participate in the in-depth interviews in a comfortable and voluntary atmosphere.

### 2.2. Study Participants

The participants of this study were 12 university students who had been continuously participating in their university Taekwondo demonstration team activities. Taekwondo demonstration activities are popular among young people, and are conducted mainly by university Taekwondo departments. Therefore, male and female students with more than two years of experience in the Taekwondo demonstration team at Baekseok University in Korea were selected as the participants of this study (Table 1).

### 2.3. Data Collection

Data collection for this study was conducted through one-to-one in-depth interviews. Interview guidelines prepared through preliminary research were used for the in-depth interviews, and the interview guidelines were modeled on individualized and semi-structured forms including standardized and open questions [29,30] (Table 2). As for the interview methods, unstructured and semi-structured interviews were conducted. The semi-structured questions in this study were verified by three qualitative research experts. This questionnaire was composed by background, exploration, and essential question types according to the argument of Berg (2004) [31].

Preliminary interviews for the preliminary research were carried out from June to July 2020, and the second and third rounds of interviews were held from August 2020 to January 2021. The preliminary interviews were conducted for 40 to 60 min per person, and the second and third interviews for 30 to 50 min. The interview locations were arranged through consultation with the study participants, and the in-depth interviews were recorded with their consent. The recorded interviews were transcribed on computer.

### 2.4. Data Analysis

The collected data were analyzed using the method of Van Kaam (1969) among the data analysis methods of phenomenological research [32]. The analysis method of Van Kaam (1969) has the advantage of being able to identify the meaningful statements in the phenomena in life experienced by study participants as well as to derive priorities according to the frequencies of statements [33]. The detailed analysis methods based on this analytic framework are as follows. First, the researcher repeatedly listened to the conversations recorded during in-depth interviews with the study participants, and transcribed all the statements of the study participants. Second, significant statements were extracted in clauses and sentences. These meaningful statements were extracted from the transcribed data in relation to the topic of this study. Third, the researcher repeatedly read the raw data extracted in the previous step to determine subthemes, collected common factors from the subthemes to determine themes, and put these themes together to categorize them.

### 2.5. Integrity of Study

In conducting qualitative research, how to ensure the reliability of data may be an important issue; therefore, in qualitative research, the terms “integrity” or “authenticity” are used instead of “reliability” to represent the reliability of qualitative data [34] and ensure the validity of the research [29]. To secure the integrity of the data, the researcher used three methods: member checking, triangulation, and peer debriefing.

## 3. Result: Cathartic Experiences in Taekwondo Demonstration Activities

### 3.1. Vicarious Purgation of Repressed Emotions

The study participants said that Taekwondo demonstration activities are a channel to release their emotional baggage created in daily life, and it was found that they experienced catharsis as their repressed emotions were relieved.

*“When I successfully perform a high-level Taekwondo skill, it feels like all the stress is blown away at once. When practicing an advanced skill, I forget everything else and only focus on that skill. Practicing Taekwondo demonstration is the only time I can relieve all my stress.”*

(Participant 1)

*“When I participate in the demonstration team practice and sweat, it feels like the impurities in my mind are cleansed… When I sweat… Then with that sweat, I feel as if my anxiety and worries are removed from my mind… I really like those moments… Soaked in sweat, I feel that I am alive, and I like that feeling…”*

(Participant 2)

In addition, Participant 12 mentioned that activities in his Taekwondo demonstration team seem to have the effect of vicarious purgation of emotions or desires.

*“Taekwondo demonstration shows are held in a very heated atmosphere. Strong energy is expressed in the form of music, dance, wood breaking demonstration, etc. It seems that a Taekwondo demonstration show is an outlet of youthful energy. During the show, I am the protagonist of the show, and it feels like good energy springs up inside me.”*

(Participant 12)

Catharsis, as a highly medical term, means the removal of the accumulated debris in the human body and the cure of diseases. Aristotle, who was deeply interested in physiology, applied this term to emotional life [35]. Therefore, the term catharsis can be understood in the sense of purifying the mind by purging any repressed, accumulated desires or emotions in the human subconscious.

**Figure 1:**
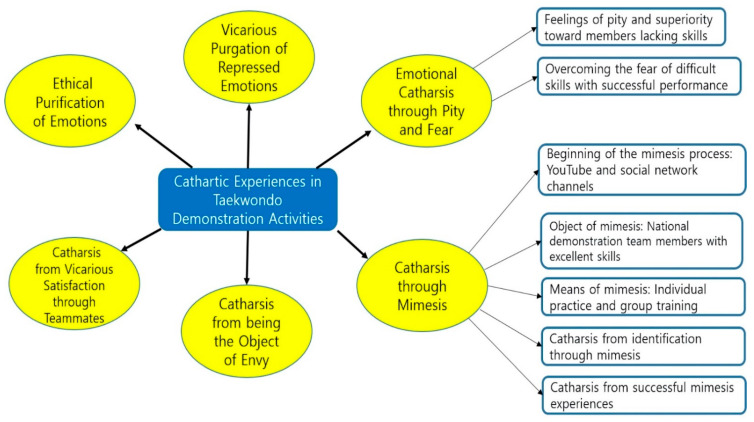
Result diagram.

Figure 1 is a diagram of the research result.

### 3.2. Emotional Catharsis through Pity and Fear

The hypothesis that the vicarious purgation of emotions occurs through the feelings of pity and fear and that this leads to the experience of catharsis was found to be valid for Taekwondo demonstration team activities.

#### 3.2.1. Feelings of Pity and Superiority toward Members Lacking Skills

In the interviews, members of the Baekseok University Taekwondo demonstration team said they usually have a feeling of pity toward teammates who are less skilled than them. They also feel a sense of superiority to these less skilled members when helping them master Taekwondo skills, which leads to the experience of catharsis.

*“I feel sorry to see my teammates who lack some Taekwondo skills striving to acquire the skills. So, I help them master those skills. I also feel a sense of superiority when these less skilled members look at me demonstrating with envious eyes.”*

(Participant 11)

*“Many of my teammates want to perform kicks and flips just as well as I do. When my teammates cheer for my demonstrations, I feel a sense of superiority for some reason.”*

(Participant 3)

These interview results show that the emotion that the study participants have toward less skilled teammates is pity. This feeling of pity is accompanied by a sense of superiority that they are better demonstrators, which gives them catharsis. It is considered that a healing process takes place in sports activities as instincts repressed in daily life are released through the action of the feeling of superiority.

#### 3.2.2. Overcoming the Fear of Difficult Skills with Successful Performance

Considering that humans are also a species of animals that live under the law of survival of the fittest, it is uncontroversial that the feeling of “pity” that the strong has toward the weak and the feeling of “fear” that the weak has toward the strong are the most basic emotions underlying human existence [36]. Even in sports activities, these feelings of pity and fear are inevitably felt and expressed.

**Figure 2:**
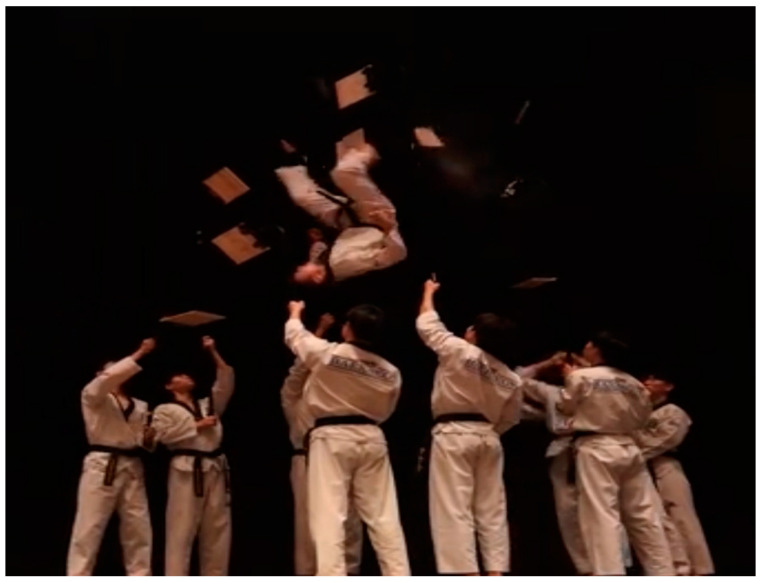
Participant’s successful spin-breaking scene in 18 December 2020.

According to the interview results, the study participants felt various emotions such as nervousness with fear, anxiety, and worry about failure when they were assigned difficult skills for a demonstration show. However, it was found that the action of emotions when they successfully performed the difficult skills had the strongest cathartic effect. When they overcame their fear and succeeded in demonstrating the skills, they felt as much joy as the fear that they first had. Figure 2 shows Participant 4’s spin breaking in the Taekwondo demonstration in December 2020.

*“At my first demonstration show in 2020, I was in charge of wood breaking with a 720° spinning kick. I practiced a lot before the performance. I succeeded in performing the skill during practice, but I was very worried about whether I could do well at the demonstration show. When my turn approached, I was overwhelmed by the fear of failure. However, I took a deep breath, gave a shout for concentration, and successfully smashed the board. The joy I felt at the moment was something that I had never experienced before.”*

(Participant 4)

*“I usually demonstrate wood breaking with ten consecutive turning back round kicks. It takes speed to perform the skill, so the teamwork with the members holding the pine boards is very important. If you miss the right timing, the boards will not break. When I smash all the ten boards in a row, one by one, I feel an excitement that cannot be described with words. It feels amazing to hear the cheers of the audience after overcoming the moments of nervousness and fear.”*

(Participant 5)

### 3.3. Ethical Purification of Emotions

The study participants also stated that they experience moral emotional catharsis that involves courtesy, respect, and a sense of fellowship toward other team players after completing a Taekwondo demonstration contest.

*“At a demonstration contest, we engage in a very fierce competition with other teams. Each team does their best to win. This, however, does not mean that we want the opposing team to fail when they perform wood breaking or other skills. Instead of wanting the other teams to make mistakes, we need to win the competition by our own ability. After the competition is over, regardless of the result, we show respect to each other. I do think that members of the other teams are also my fellow Taekwondo demonstrators.”*

(Participant 9)

*“In a sparring fight, we have a one-on-one physical fight with the opponent, but in a demonstration contest, it is the skills that each team demonstrates and the composition of their performances that determine the winner. That is why teamwork and harmony are important in a demonstration contest. So, at the end of the contest, a solid bond is formed among the teammates who practiced together for the demonstration. As we know that the demonstrators of other teams also worked hard to prepare for the contest, we can also cheer for them.”*

(Participant 8)

As modern sports become commercialized and triumphalist, unethical practices in the sports world often emerge as social problems [37]. However, the catharsis experienced by the participants of this study can be seen as the ethical purification of emotions through sports activities. This is an elevation of moral emotions through courtesy and consideration toward rival teams and respect and a sense of fellowship with competitors.

### 3.4. Catharsis through Mimesis

It was found that the study participants experience catharsis through successful mimesis experience and identification with the object of mimesis. The following is the process of mimesis experienced by the Taekwondo demonstration team members.

#### 3.4.1. Beginning of the Mimesis Process: YouTube and Social Network Channels (Instagram, Facebook, etc.)

For the study participants, the mimesis process began with watching Taekwondo athletes demonstrating high-level wood breaking skills through YouTube or social network channels. The mimesis process started as they became anxious to perform these difficult skills as perfectly as the athletes did.

*“These days, I look up Taekwondo demonstration a lot on YouTube. I try to identify the skills featured in demonstration videos and imitate the skills to master them.”*

(Participant 7)

*“I am following famous Taekwondo athletes who have excellent wood breaking skills on Instagram and Facebook. I try to imitate their skills watching the demonstration videos posted on their Instagram account.”*

(Participant 6)

Currently, it is easy to find information on various Taekwondo wood breaking skills on YouTube and social network channels. In many of the study participants, the mimesis process began with the search for information related to Taekwondo demonstration on YouTube or social network channels.

#### 3.4.2. Object of Mimesis: National Demonstration Team Members with Excellent Skills

To the study participants, the object of mimesis was top-ranked members of the national Korean Taekwondo demonstration team who execute the skills they want to demonstrate. When the study participants succeeded in performing the skills demonstrated by the object of mimesis, they identified themselves with these top-tier Taekwondo athletes, which provided catharsis for them.

*“Athlete 000 of the national demonstration team commands the best jump skill in Korea. I practice hard to imitate the skill. When I succeed in performing the skill of 000, I feel much excited that I can perform like him.”*

(Participant 4)

*“I think Korea’s best consecutive kick demonstrator is 000. 000 is my role model. When I successfully perform the skill he demonstrates, I feel confident that I can perform like him, identifying myself with him.”*

(Participant 5)

The study participants were very interested in the skills and performance of the best active Taekwondo athletes, which had a great influence on their demonstration styles. In other words, it appears that they prefer the styles and moves of the best active Taekwondo athletes that they like.

#### 3.4.3. Means of Mimesis: Individual Practice and Group Training

Participants in this study were identified as engaging in mimesis through individual practice and group training. The study participants usually taught themselves the skills they decided to imitate through personal practice and then applied the skills they imitated to group training.

*“I teach myself the wood breaking skill of 000 during my individual practice time. Since group training takes up a large portion of the demonstration team training, I personally take time for practicing the skill that I am going to demonstrate.”*

(Participant 12)

*“I individually practice the wood breaking skills of my favorite Taekwondo athletes. When I show my teammates the skills I mastered through individual practice during group training, they compliment me and I feel flattered.”*

(Participant 1)

#### 3.4.4. Catharsis from Identification through Mimesis

The participants of this study were found to feel a great joy by identifying themselves with Taekwondo athletes they want to emulate. The Greek term “mimesis” has the meaning of “representation” rather than “copying.” In terms of physical education, mimesis can be interpreted as the representation of high-level skills [38]. The study participants mentioned that they experienced catharsis as they felt a great joy when they succeeded in reproducing the skills of top-tier Taekwondo athletes whom they admired and identified themselves with.

*“When I practice to master the skills of Taekwondo athlete 000, who is my role model, I imagine that I became the athlete.”*

(Participant 2)

*It feels great when my teammates see me practicing some skills and tell me that my moves remind them of those of Taekwondo athlete 000, who is my idol.*

(Participant 9)

In psychology, mimesis is defined as a phenomenon where one reproduces or performs an action of another person or an animal that stimulated one in an identical or similar form [39]. The study participants experienced the joy of psychological identification and the resulting catharsis through the process of identification with the athletes they want to emulate by reproducing and performing their actions.

#### 3.4.5. Catharsis from Successful Mimesis Experiences

It was found that the study participants’ experience of success in reproducing the skills of the athletes they want to emulate and achieving the purpose of reproducing and executing the skills made the study participants feel catharsis regarding the accomplishment. These success experiences provide greater catharsis when they take place during demonstrations performances than during practice.

*“During my performance, I successfully demonstrated the scissors kick skill of Taekwondo athlete 000 that I had practiced very hard. Listening to the applause and cheers of the crowd, I felt a quiver of joy.”*

(Participant 8)

*“I mastered the high-level wood breaking with a flip kick skill of athlete 000 after practicing for a very long time. Finally, I had the opportunity to demonstrate that skill in a performance, and I gave a perfect demonstration though I was very nervous. It still makes me feel so happy to recall that success experience.”*

(Participant 10)

As shown in the study results [35,40], success experiences in sports activities have a positive effect on adaptive behavior and control of deviant behavior in society. The study participants reported that they felt catharsis from emotional satisfaction and skill improvement through the experience of successfully imitating the skills of the athletes they want to emulate; that is, the success experiences in sports activities.

### 3.5. Catharsis from Vicarious Satisfaction through Teammates

The study participants said that even when they play the role of demonstration assistants during a demonstration performance, they experience catharsis from vicarious satisfaction while seeing the excellent skills of their teammates. Particularly when their teammates demonstrate high-level skills that cause a tense atmosphere, they feel the same tension as the demonstrators, and after they successfully demonstrate the skills, they feel as if they performed the skills themselves.

*“I am the leader of my team, and it seems like I feel the happiness and excitement that my teammates feel each time they successfully demonstrate skills. Especially, when they give a perfect demonstration of high-level skills, I really feel thrilled. I become sensitive to every move of my teammates, get excited by it, and during a demonstration show, I feel so united with all my teammates.”*

(Participant 4)

*“In a demonstration performance held a while ago, 000 succeeded in performing the wood breaking with a flip kick that he practiced hard. At that time, I felt extremely happy as if I myself had succeeded in it.”*

(Participant 3)

This various satisfaction is psychologically referred to as substitute behavior. Substitute behavior is an action taken when a goal was not fulfilled due to a certain obstacle; one satisfies the initial needs by achieving another goal that substitutes for the first goal [41]. According to the interview results, it was analyzed that the study participants also experience vicarious purgation and catharsis while watching the excellent performances of their teammates, even when they participate in the demonstration show as assistants instead of demonstrating wood breaking themselves.

### 3.6. Catharsis from Being the Object of Envy

**Figure 3:**
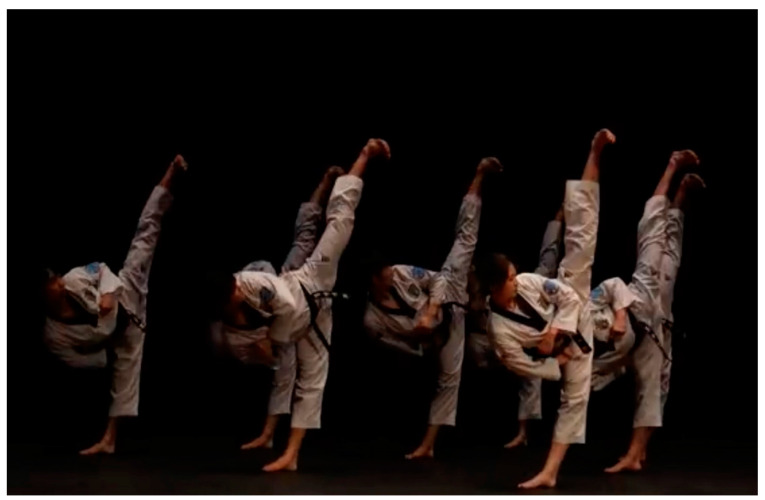
Participants’ side kick demonstration scene in 18 December 2020.

Celebrities and famous sports stars that enjoy popularity among the public often become the object of envy by others. The public emulate them as the object of mimesis. These public attentions make celebrities experience the catharsis that comes from being the object of envy. In this study, it was analyzed that Taekwondo demonstration team members also experience catharsis that comes from becoming the object of envy of their teammates or audiences with their wonderful performances. Figure 3 shows participants kicking during a demonstration performance.

*“Not long ago, I performed at a demonstration show held to celebrate a Taekwondo competition. After the demonstration was over, children asked me to take pictures together. They said that my demonstration was great and that my skills were very cool. I felt catharsis in that I became the object of someone else’s envy.”*

(Participant 1)

*“When I demonstrate Taekwondo skills, I feel like people are looking up at me. When I hear the cheers of the crowd during my performance, I feel like I have become a celebrity.”*

(Participant 5)

Taekwondo demonstrators feel as if they have become the object of envy through their wonderful demonstration. It turned out that they experience catharsis that comes from being the object of envy through the reaction of the audience who are fascinated by their good looks and demonstration.

## 4. Discussion

Based on the analysis of the collected data, the demonstration team members’ cathartic experiences were largely divided into six categories.

First, the study participants experienced catharsis from vicarious purgation of repressed emotions. For them, the university Taekwondo demonstration team activities served as a channel to release the emotional baggage that had been repressed in their daily lives, and they experienced catharsis through these activities. The catharsis that Aristotle discussed is not the “repression” of emotions, but the “purgation” of emotions [42]. Plato stressed that repressing emotions as much as possible and restoring calm reason is helpful in realizing the idea, the real world. However, contrary to Plato, Aristotle believed that in order to get rid of confusions in rational life, it was necessary to properly express and release emotions [43,44]. The interviews with the participants of this study showed that Taekwondo demonstration is not just an act to strengthen physical strength, but also an action of mental purification through the purgation of emotions.

Second, they experienced emotional catharsis through pity and fear. They felt a feeling of pity for their teammates who lacked demonstration skills, which gave them catharsis, allowing them to have a sense of superiority. They also experienced the catharsis of success when they overcame their fear of difficult demonstration skills by successfully performing them. Regarding pity and fear, Aristotle explained in the 13th chapter of the Poetics that pity arises when one sees a protagonist unfairly fall into misery, while fear is generated when one sees a protagonist that he can relate to fall into misery [44].

Third, the Taekwondo demonstrators experienced catharsis from ethical purification of emotions. They experienced the catharsis of ethical and moral feelings when they finished Taekwondo demonstration contests and expressed courtesy and respect for other teams and when they formed strong bonds with their teammates. Catharsis as a mental purification process can be approached from the ethical aspect of sports. Soccer players fiercely and separately compete with the other team based on the spirit of fair play throughout the game, 45 min for each half, but after the game, they exchange their uniforms with their opponents, shaking hands and hugging each other. The spectators who see this and the players themselves feel joy and experience moral catharsis about the fair and ethical completion of the match and the considerate attitude toward each other, regardless of the result of the competition [15].

Fourth, they experienced catharsis through mimesis. The process of catharsis through mimesis is as follows. Major means to perform mimesis were identified as YouTube and social network channels. The object of mimesis was the top-tier Taekwondo athletes in the national demonstration team. Mimesis occurred mainly through individual practice and group training, and it was analyzed that the students felt catharsis from identification of themselves with the object of mimesis and catharsis through successful mimesis. The Romans translated the Greek word “mimesis” as “imitaio” (“imitation” in modern French and English), and in the East, “imitaio” has been commonly translated into words meaning “imitation.” However, while the word “imitation” has relatively strong connotations of “fake” and “counterfeit”, “mimesis” is a word less connotative of these concepts; therefore, some scholars use the word “mimesis” instead of “imitation” or translate it as “representation.” [45]. In addition, according to Aristotle, mimesis includes poetry, painting, sculpture, and even music and dance. He saw that the music played by musical instruments such as the flute, lyra, and reed pipe, as well as dance, is a sort of mimesis of human personality, emotions, and actions. Therefore, the meaning of mimesis can be extended to a concept that is represented by the word “expression” today [46].

It can be said that mimesis is a process that anyone goes through in sports activities, and this process allows us to learn different skills and enjoy sports. In his theory of catharsis, Aristotle noted that humans reach a cathartic experience through the process of mimesis [35].

Fifth, the study participants experienced catharsis from vicarious satisfaction through teammates. Even if they participated in demonstration performances as assistants, they experienced catharsis from vicarious satisfaction while seeing the excellent skills of their fellow demonstrators. Catharsis refers to the vicarious purgation of accumulated or repressed emotions. According to Aristotle, the repressed psychological state makes the audience feel pity and fear as they see the protagonists in a play or art work falling into a tragic situation, through which emotional catharsis is experienced in the audience [47]. In other words, Aristotle saw that audiences experience the vicarious purgation of emotions or desires as they relate to the situation on the stage [48]. Even in sports settings, people can experience catharsis through vicarious satisfaction as the audience or fellow players [49]. During the 2002 World Cup, the Korean team achieved a feat of advancing to the semifinals, which made all Koreans who were the audience of the games experience the catharsis of victory and joy. The whole nation seemed to feel proud of being called “Red Devils”, the name of the official supporting group of the Korean national soccer team, and identified themselves with the national players as if becoming the 12th player on the ground [50].

Lastly, they experienced catharsis from being the object of envy. When they gave excellent performances, they felt catharsis based on self-satisfaction that they became the object of envy by others. They also experienced the catharsis that comes from being the object of envy through the reaction of the audience fascinated by their good looks and wonderful performances.

This study rediscovered the spiritual value of Taekwondo practice based on the understanding of the characteristics and meanings of catharsis experienced through Taekwondo demonstration team activities. Most studies on mental health in Taekwondo have shown that it helps with concentration, confidence, and self-esteem through Taekwondo training [5,6,7,8,9,10,11,12]. However, this study suggests that Taekwondo training can help improve mental health through the experiences of various forms of catharsis as presented in the results.

Many studies have been conducted on the mental utility of sports activities. Catharsis essentially refers to a release or discharge or cleansing of emotions, generally with the purpose of relieving the stress that develops from holding these emotions within the individual. We often refer to the cathartic nature of sport (and exercise), relieving stress or tension that might build up, or serving as a release for anger and hostility [49].

The concept of catharsis, used by Freud for the purpose of psychotherapy based on the theory of Aristotle, contains the principle that can be approached from the perspective of healing. Freud noted that when repressed anger accumulates inside, symptoms such as hysteria, anxiety, and frustration can increase rapidly [51]. Miller et al. (1939) saw that repressed aggression can result in frustration and further increase in aggression [52]. Buss observed that a violent act of aggression could provide satisfaction through the expression of the action and lower the level of anger [53]. In addition, Berkowitz noted that a person in anger can have a feeling of satisfaction when he expresses it as if he fulfilled his duty [54].

The psychotherapeutic approach based on catharsis serves as the foundation for the hydraulic model of anger [55]. According to this model, dissatisfaction or frustration turns into anger, and that anger continuously accumulates in the individual’s mind. The anger is compared to hydraulic pressure trapped in a space disconnected from the surrounding environment, and remains in this state about to burst until it is released in any way. The anger accumulated inside will eventually burst when not released, resulting in aggressive anger. In other words, repressing anger can have a poisonous effect. The modern theory of catharsis from the therapeutic perspective focuses on the effective expression and mitigation of the inner repressed anger that is generated from the feelings of anger based on this model.

The use of catharsis from this therapeutic perspective focuses on mitigating aggressive behavior in conditions such as anger or frustration. From this viewpoint, expression of emotions can be used as a context for healing. Modern people remove unmet needs in reality through sports, which are characterized by physical activity, interactivity, success experiences, and vicarious satisfaction. Sports activities provide a sense of psychological healing by resolving dissatisfaction and violent psychology accumulated inside through the free expression of emotions [56,57,58,59,60,61].

Research with students has demonstrated that high-quality Taekwondo training can improve children’s self-regulation, executive function, behaviors, and motor skills. Affective benefits of Taekwondo training include increasing one’s sense of self-efficacy, self-esteem, emotion regulation, and resilience. Taekwondo training provides opportunities for children to make progress in small increments like learning a simple form or kick, thus creating opportunities for small successes [62].

The university Taekwondo demonstrators who participated in this study relieved the stress experienced in modern society, felt ethical emotions, and experienced various feelings of satisfaction through the practice of Taekwondo. These findings confirm that Taekwondo, a traditional Korean martial art, is a sports activity that allows people to experience catharsis, which is a mental effect of sports.

This study adopted phenomenological research methods for the in-depth understanding of catharsis experienced by university Taekwondo demonstrators. Based on the study findings and limitations revealed in the study process, the issues needed to be considered in follow-up studies are suggested as follows.

First, the subject of this study was limited to the field of Taekwondo demonstration. Cathartic experiences in the fields of Taekwondo pumsae (sequences of movements) and gyeorugi (sparring fights) can be good research topics. Second, despite the COVID-19 pandemic, golf courses in Korea are still very crowded, and golf remains a popular sport. As a follow-up study, a study on catharsis experienced in golf is proposed.

## 5. Conclusions

In conclusion, the results of this study suggest that the catharsis of Taekwondo training is a good tool to solve various mental problems in modern society. The essential meaning and utility of catharsis suggested by Aristotle also appeared in the activities of the Taekwondo demonstration team.

Taekwondo training has various functions. Among them, the catharsis function of Taekwondo training in this study will further expand Taekwondo.

Modern people relieve mental and physical stress through sport activities. Therefore, the cathartic function of sports will be further expanded.

## Figures and Tables

**Table 1:** Characteristics of participants.

ID	Gender	Age	Demonstration Team Career	Taekwondo Grade
Participant 1	M	23	5 years	4th Dan
Participant 2	F	22	5 years	4th Dan
Participant 3	M	21	6 years	3rd Dan
Participant 4	M	22	3 years	4th Dan
Participant 5	F	23	4 years	4th Dan
Participant 6	M	21	4 years	4th Dan
Participant 7	M	24	6 years	5th Dan
Participant 8	M	24	5 years	5th Dan
Participant 9	F	20	1 year	4th Dan
Participant 10	F	21	4 years	4th Dan
Participant 11	M	24	5 years	5th Dan
Participant 12	M	25	5 years	5th Dan

The term Dan is commonly used in Taekwondo to denote a black belt. Black belt begins from 1st Dan. It denotes a grade or level. Dan is made up of nine grades.

**Table 2:** Semi-structured interview questionnaire.

Interview Question
Could you tell me about your Taekwondo demonstration team activities? When was the happiest moment during Taekwondo demonstration team activities? Have you ever experienced any stress relief or catharsis during Taekwondo training or demonstration performance?

## Data Availability

The data presented in this study are available on request from the corresponding author.
